# The Mediating and Buffering Effect of Creativity on the Relationship Between Sense of Place and Academic Achievement in Geography

**DOI:** 10.3389/fpsyg.2022.918289

**Published:** 2022-06-21

**Authors:** Jianzhen Zhang, Jiahao Ge, Yuting Ma, Ziyang Wang, Yuyao Yu, Xiaoyu Liang, Zhenni An, Yanhua Xu

**Affiliations:** College of Geography and Environmental Sciences, Zhejiang Normal University, Jinhua, China

**Keywords:** sense of place, creativity, academic achievement in geography, mediating and buffering effects, upper-secondary-school students

## Abstract

**Purpose:**

This study explored the relationship between sense of place and academic achievement in geography and used a mediation model to verify the mediating role of creativity in this relationship.

**Methods:**

A total of 1,037 upper secondary school students were surveyed using the *Sense of Place Scale*, the *Innovative Behavior Scale*, and their *geography test scores*. SPSS (version 26.0) was used for descriptive statistical analysis and correlation analysis. The PROCESS plug-in (version 4.0) was used to test the mediating effect of creativity.

**Results:**

(1) The correlation analysis showed that sense of place has a positive effect on academic achievement in geography and is related to creativity. Moreover, creativity exerts a positive effect on academic achievement in geography (2). The results of mediation analysis indicated that creativity plays mediating and buffering roles in the relationship between sense of place and academic achievement in geography after controlling for gender and residential address. The direct and indirect effects accounted for 65.708 and 34.292% of the total effect, respectively.

**Conclusions:**

The results indicated that sense of place affected not only academic achievement in geography directly but also creativity indirectly. This conclusion provides certain ideas for the development of geography curriculums. Since academic achievement in geography is related to both sense of place and creativity, it is necessary to pay more attention to integrating sense of place in geography education and to foster creativity in curriculum development and teaching of geography.

## Introduction

Geography, as one of the core subjects, is incorporated into the Framework for twenty-first century learning, relating to the development of twenty-first century skills (Sugiyanto et al., 2018; Liu et al., 2022). Academic achievement in geography is a result of what children and young people know and are able to do after learning geography (Solem et al., 2018). The GeoCapabilities project, funded by the European Union's Comenius Program, associated it with individuals' imagination, thinking, and reasoning (Lambert and Jones, 2017). Also, improvement in academic achievement in geography can promote students to critically and creatively solve problems (Sugiyanto et al., 2018). Although geography would be absent in the NAEP assessment schedule through 2029 (Solem and Stoltman, 2020), academic achievement in geography has still been regarded as indicators of progression in geographic literacy of American students (Dolan, 2016; Solem and Stoltman, 2020). Moreover, it is of great significance to cognitive and emotional development and closely related to overall academic achievement, playing a key role in all-round personal growth.

Recently, influences on academic achievement in geography have drawn some researchers' attention. Individual factors, such as gender, learning attitudes, and self-efficacy, have been linked to it (Chambers and Schreiber, 2004; Filgona and Sababa, 2017; Ansong et al., 2019). Also, researchers have found that external factors like residential address, teacher quality, and instructional strategies have impacts on academic achievement in geography (Okafor, 2016; Zboun et al., 2018). Environment is another factor related to academic achievement in geography. For instance, a warm family environment can enhance it (Mustika et al., 2021), and other research has identified potential associations between the classroom environment and academic achievement in geography (Bondarenko, 2018). In addition, outdoor learning environments can exert a more positive effect on academic achievement than an indoor class (Hawa et al., 2021).

Sense of place, which is defined as a combination of the environment and perception (Tuan, 1975), represents complex feelings toward a specific place, like school or hometown (Quinn et al., 2019). Recently, the relationship between sense of place and geography education has aroused interest of researchers (Varró and Van Gorp, 2021; Tan, 2022). Sense of place has been proved to be an important element in geography education (Preston, 2015), affecting the development of academic achievement in geography (Hooykaas, 2021). In addition, creativity has also been connected to academic achievement in geography: Catling (2005, 2013) argued that creativity is an essential element of the study of geography, and others have similarly confirmed that the level of creativity directly affects students' academic achievement in geography (Hintermann et al., 2020).

Although researchers have not previously focused on the complex relationships between sense of place, creativity, and academic achievement in geography, certain studies may provide relevant evidence of their interactions. First, according to the 4P model (Rhodes, 1961) and the investment theory of creativity (Sternberg and Lubart,1991), creativity is closely connected to the environment and perception. The mechanism of how the environment and perception influence creativity has attracted the attention of many researchers, but few have examined the relationship between sense of place and creativity. Furthermore, the environment and perception definitely have impacts on academic achievement (Khudadad and Mickelson, 2021); however, it has not been proven that sense of place is directly correlated with academic achievement in geography. To sum up, sense of place, creativity, and academic achievement in geography may be interconnected, but the mechanisms are unclear and merit further investigation.

Therefore, to enhance comprehension of the role of sense of place in geography education, we explored the relationship between sense of place and academic achievement in geography along with the role of creativity in mediating between the two. Gender and residential address were considered as covariates and were controlled in the process. In the next section, the definition of the three constructs, their influencing factors, and the relationships between them are discussed.

## Theoretical Basis and Hypothesis

### Sense of Place

In the 1970s, Lowenthal (1972) and Tuan (1975) introduced the concept of sense of place, which was defined as both the inherent characteristics of a place and the complex connections that people build with it. These connections have cognitive, behavioral, and emotional dimensions (Nelson et al., 2020), although sense of place had been more often regarded as an emotional connection between humans and place (Kastenholz et al., 2020), or a feeling toward a specific place, like school and hometown (Quinn et al., 2019). Concepts, such as place dependence (PD), place attachment (PA), and place identity (PI), can be considered as subordinate concepts of sense of place (Shamai and Ilatov, 2005; Gillespie et al., 2022). PD refers to a dependence on a place in order to achieve certain goals and do things that we want to do (Pretty et al., 2003). PA is defined as the emotional bonds that people have developed with places (Proshansky et al., 1983). PI is linked with the process where personal identity emerges out of affective and social relations with places (Williams, 1989).

A place is the foundation of human existence, providing the resources for survival and also bringing a sense of security and shelter (Tuan, 2011). Sense of place is regarded as a combination of the environment and perception: interactions between humans and places produce the sense of place (Relph, 1976; Steele, 1981). Relph (1976) argued that sense of place is related to the elements of the physical and social environment of a place and that the environment provides the necessary material basis for the formation of sense of place and the attachment of meaning to it. There is also a link between sense of place and perception. Tuan (1990) proposed the concept of *topophilia*, indicating that perception and experience play important roles in development of cognition to a place, and that sense of place can be viewed as what individuals construct by making full use of their multisensory perceptual abilities (Tuan, 1990; Raymond et al., 2017). Moreover, Relph (1976) stated that sense of place is a special human–place relationship based on the real experience and real emotion of people in that environment. In recent years, evidence from neuroscience has shown that embodied elements, such as behavior, perception, and emotion, are related to the formation of sense of place (Lengen and Kistemann, 2012; Campelo, 2015; Raymond et al., 2017).

The literature has shown a trend toward diversification of the measurements of sense of place. Qualitative research methods, such as in-depth interviews, participant observation, and videotaping, have a major part to play (Amsden et al., 2010; Cunsolo Willox et al., 2012; Wartmann and Purves, 2018; Sebastien, 2020). In recent years, mixed research methods have been growing in popularity (Deutsch and Goulias, 2012; Friesinger et al., 2019). Scales based on a conceptual model are the most widely used measurement method (Shamai, 1991). Consequently, our study adopted the model of sense of place and the measurement instrument developed by Jorgensen and Stedman (2001), who divided sense of place into three dimensions (PD, PA, and PI) and developed a 12-item scale.

### Academic Achievement in Geography

*Academic achievement* refers to the comprehensive performance of knowledge, skills, and competencies acquired by students after learning a certain subject (Gage et al., 2015; Boonk et al., 2018). *Academic achievement in geography*, in a broad sense, is the combination of knowledge, skills, emotions, attitudes, and values acquired by completing courses in geography (Were, 2011; Hursen and Beyoglu, 2020). In this study, academic achievement in geography is defined as standardized test scores (Turan et al., 2018).

Academic achievement in geography is influenced by many factors, for instance, the application of geographic information technology, the use of social media platforms, such as YouTube, and the style of illustrations in geography textbooks (Aladag, 2010; Çifçi, 2016; Estawul et al., 2016; Zboun et al., 2018). Likewise, the geographic literacy of geography teachers can generate a significant effect on students' academic achievement in geography (Filgona and Sakiyo, 2020), and individual factors, such as gender, interest in learning, and self-efficacy, have varying impacts (Filgona and Sababa, 2017). Many studies revealed that academic achievement in geography is associated with gender (Filgona and Sababa, 2017; Herrera et al., 2020; Makowsky and Martin, 2021). Solem et al. (2021) stated that there is a statistically significant relationship between gender and academic achievement in geography in each assessment year based on the data from NAEP. However, few available studies have focused on the connection between sense of place and academic achievement in geography (Marvell and Simm, 2016).

The environment has been linked to academic achievement in geography, with some studies confirming that a cohesive climate in a geography classroom significantly increases academic achievement in geography (Chionh and Fraser, 2009; Bondarenko, 2018). Similarly, a comfortable home environment with a full range of amenities has been identified as a positive factor in academic achievement in geography (Sarma et al., 2016). In addition, blended learning environments and virtual classrooms have been viewed as elements that can improve academic achievement in geography (Dikmenli and Ünaldi, 2013). As for the residential address noted, several studies have highlighted the significant impact of it on academic achievement in geography (Sivak et al., 2017; Boateng et al., 2021). Differences in region and urbanicity have been proved to exert significant influences on academic achievement in geography (Solem et al., 2021).

Moreover, perception is considered as an important influencing factor. Students who perceive the school climate, social status, and emotions of others positively usually achieve academic success (Sarwar and Tarique, 2016). Likouri et al. (2017) showed that the better students' spatial and environmental perception, the better their performance in geography. Furthermore, there is growing evidence that emotion and affection lead to changes in students' academic achievement. Positive affects, including feelings, emotion, and even love, can promote students' academic efficacy in geography (Downey et al., 2008; Ye et al., 2021c). In addition, the interaction between emotion and intelligence has been proved to enhance academic achievement in geography (Hill et al., 2021). As previously mentioned, sense of place is a combination of the environment and perception and an emotional connection between humans and places (Tuan, 1990). Based on the literature review, which shows that environment, perception, cognition, and emotion are all important factors in academic achievement in geography, we hypothesize as follows:

Hypothesis 1: Sense of place positively affects geography achievement in geography.

### Creativity

Guilford (1950, 1967) defined *creativity* as the ability to produce novel and applicable ideas or products, with the two main characteristics being novelty and validity. Humanistic psychologists have identified two paths that promote creativity: leading individuals to develop more creative self-awareness and improving the individuals' environment so as to stimulate their creative expression (Woodman, 1981). Gestalt psychologists have suggested that the enhancement of creativity includes preparation, gestation, clarification, and validation (Smith, 2015) or the five steps of problem representation, gestation preparation, generation of response, validation screening, and evaluation of results (Amabile, 1983). Currently, the conclusion that creativity is related to cognitive processes is universally accepted (Runco and Basadur, 1993; Xu et al., 2021; Zeng et al., 2021). Creativity has also been conceptualized as divergent thinking, creative problem solving, and other forms of intellectual performance (Kaufmann, 1988; Fredrickson and Branigan, 2005; Zabelina and Ganis, 2018; Gralewski and Karwowski, 2019; Frith et al., 2021).

When exploring the relationship between the sense of place and creativity, it is necessary to focus on several classic theories about creativity. First, the *4P model of creativity* (Rhodes, 1961) integrates four elements related to creativity: person, process, product, and place. The *componential theory* proposed by Amabile (1983, 1996), one of the most important theories of creativity, highlights the impact of cognitive, personality, motivational, and social factors. Later, she updated the model to add social environment (Amabile, 1997). Sternberg and Lubart (1991) proposed the *investment theory* of creativity, which considers creativity as the result of the interaction of the individuals, their environment, and the specific task. The core idea of this theory is that creativity is influenced by intellectual processes, knowledge, intellectual style, personality, motivation, and environmental context. Kaufman and Beghetto (2009) propounded the *Four-C model* of creativity, which includes *mini-c* (creating novel and personally meaningful interpretations, experiences, behaviors, products, or events), *little-c* (innovative behaviors in a familiar context), *pro-c* (innovative behaviors in a specialized domain), and *big-C* (innovative behaviors that make a significant contribution). Hence, we find that these classic theories fit more or less well with our study of the connection between sense of place and creativity.

Numerous studies have been concerned with the factors that influence creativity. Individual factors, such as gender, cognition, emotion, motivation, and efficacy, have an impact (Kwasniewska and Necka, 2004; Dewett, 2007; Hennessey, 2010; Schmidt, 2011; Xu et al., 2021). Particularly, the topic of gender differences in creativity generates substantial scientific and public interest (Abraham, 2016). Also, external factors, including environmental factors, have been connected to creativity (Drake, 2003; Erez and Nouri, 2010). In our comprehensive review of the classic theories of creativity listed before, we found that creativity is related to the environment, perception, and emotion. A study revealed that the residential address has an effect on creativity (Gong et al., 2019), and another one further stated that proximity to a natural environment enhances creativity (Plambech and Konijnendijk van den Bosch, 2015). In addition, several studies have demonstrated that a positive environment can promote creativity (Richardson and Mishra, 2018; Arslan et al., 2022), and positive perceptions also significantly strengthen it (Kwasniewska and Necka, 2004; Xu et al., 2021). Moreover, the generation and development of creativity can be influenced by positive emotions (Radford, 2004; Conner and Silvia, 2015; Ivcevic and Hoffmann, 2019; Kung and Chao, 2019; West and Somer, 2020).

However, little is known as yet about the relationship between sense of place and creativity. As demonstrated previously, the available evidence shows that positive environmental, perceptual, cognitive, and affective factors have a positive impact on creativity. Given that sense of place has been characterized as a combination of the environment and perception (Tuan, 1975) and the emotional connection between humans and places (Steele, 1981), there may be an intrinsic link between sense of place and creativity. Therefore, we contend as follows:

Hypothesis 2:Sense of place positively affects creativity.

To date, the relationship between creativity and academic achievement has received much attention from the scholarly community. Guilford (1967) argued that creativity and learning are essentially the same phenomenon. Similarly, creativity is considered essential for almost all human mental activities (Vygotsky, 1967). Indeed, extant research suggests that creative people can adapt to available experiences in novel ways (Sternberg, 2012). Beghetto and Kaufman (2014) contended that learning is the process of creation, involving reorganization and innovation of existing experiences and previous knowledge. In addition, some studies have reported that creativity equips individuals with a solid foundation of knowledge and skills (Wallach, 1985; Fink et al., 2010; Hanif et al., 2019; Supena et al., 2021). The Four-C model of creativity (Kaufman and Beghetto, 2009) is an important theoretical foundation for this study. The mini-c represents the creativity inherent in the learning process (Beghetto and Kaufman, 2007). This theory has also led researchers to pay more attention to students' distinct and personally meaningful insights, strengthening the connection between creativity and learning. In particular, mini-c had been closely correlated with the learning process, and evidence suggests that students with more mini-c usually achieve better academic performance (Kaufman and Beghetto, 2013). In recent years, an empirical research study (Doleck et al., 2017), along with a meta-analysis (Gajda et al., 2017), has largely supported the positive impact of creativity on academic achievement. Although few studies have examined the link between creativity and academic achievement in geography, researchers have investigated the role of creativity in geography education. For example, Scoffham (2013) stated that trying to make sense of the world and understanding the forces that act upon it require us to think creatively. Also, geography is considered as an active discipline, and creativity is at its core (Catling, 2005; Hintermann et al., 2020). In addition, other researchers have revealed that students with higher creativity perform better in inquiry-based learning in geography (Walkington et al., 2011; Tomčíková, 2020). On the basis of these arguments, we formulate the following hypothesis:

Hypothesis 3:Creativity positively affects academic achievement in geography.

Taking into account the aforementioned predictions, we predict that sense of place is associated with creativity, which in turn predicts academic achievement in geography, so we hypothesize as follows:

Hypothesis 4:Creativity plays a mediating and buffering role between sense of place and academic achievement in geography.

**Figure 1:**
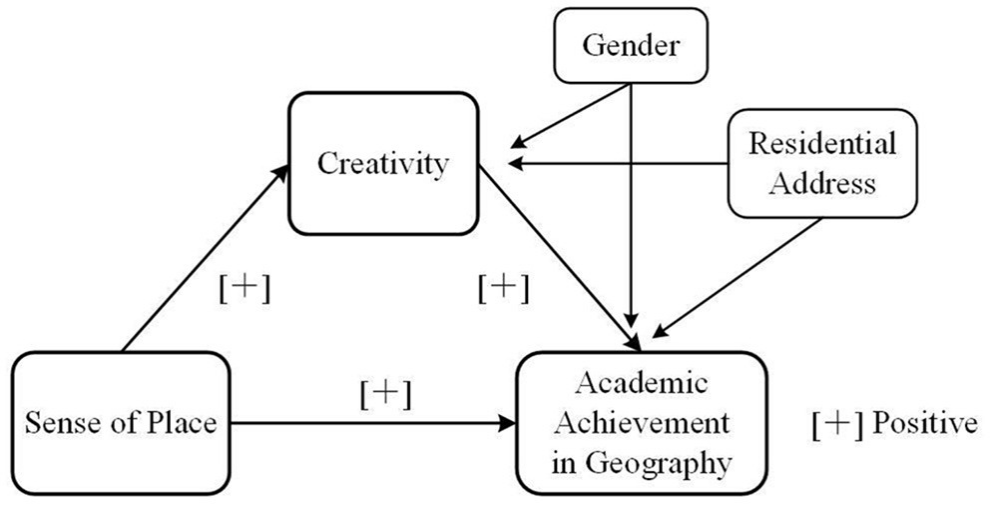
Relationships examined in the study.

Figure 1 depicts the mediation model proposed in the four hypotheses, showing the relationships between the independent, mediator, and dependent variables and two covariates.

## Materials and Methods

### Participants and Procedures

To develop ideas and hypotheses, we conducted an exploratory focus group interview in schools before finalizing the study design. Most of the interviewees said that they have a feeling of dependency on their hometown. Many said that the current surrounding environment definitely influenced their school work, although few mentioned the role of creativity in their geography learning.

We collected data in public upper secondary schools in western China. A total of 1,268 students aged 16–18 years completed the survey questionnaire between November 10 and November 30, 2021. Before the students filled out the questionnaires, we explained the study to their parents, head teacher, and geography teachers, and we obtained consent from the students and their parents. During recess, we distributed paper questionnaires to students. We collected the questionnaires after students had completed them and entered the results. The data were digitized for further study. After removing any incomplete responses, the number of valid questionnaires was 1,037.

### Materials

The questionnaire used in this study comprised three sections: demographic information, the *Sense of Place Scale*, and the *Innovative Behavior Scale*. Demographic information included gender and residential address. In addition, with the consent of both teachers and students, we obtained students' recent mid-term geography scores. Since we collected data in different schools, scores for academic achievement in geography were calculated by classifying each grade in one of six categories: grades of 90 or above, 80–89, 70–79, 60–69, 40–59, and below 40 were classified as Levels 6, 5, 4, 3, 2, and 1, respectively. In this way, students' academic achievements in geography were outlined. To ensure that participants recalled their grades, we communicated with geography teachers to ensure that the questionnaires were administered 1 week after the mid-term examination.

Totally, two scales originally developed in English were translated into Chinese for the present study. Back-translation was used to increase the accuracy of the translation (Brislin, 1970), that is, one researcher translated the instrument from English to Chinese, then a second researcher translated the Chinese version back to English, and finally a third researcher examined the equivalency of the original English version and the translated Chinese version by comparing the three versions of the instrument (original, translated, and back-translated). Any inequivalence was addressed before data collection.

### Sense of Place Scale

Adopted from Jorgensen and Stedman (2001), the *Sense of Place Scale* comprises three dimensions: place dependence, place attachment, and place identity, in line with our focus on personal feelings and emotions related to a specific place. It consists of 12 items, measured on a five-point Likert scale: from 1 point (strongly disagree) to 5 points (strongly agree). Sample items include “This place, which is relevant to me, is a reflection of my existence” and “This place is my favorite place.” After discussion, some of the item statements were modified to fit the language habits and life experiences of secondary school students. In this study, the internal consistency coefficient of the scale was 0.688, and the KMO value was 0.816.

### Innovative Behavior Scale

Adopted from Janssen (2000), the *Innovative Behavior Scale* comprises three dimensions: idea generation, idea promotion, and idea realization, focusing on the process where people generate creative ideas and practice creative behaviors. The scale consists of nine items (e.g., “I often come up with new ideas when I encounter problems”), measured on a five-point Likert scale, ranging from 1 (never) to 5 (always). Finally, the average score was calculated, and the higher the score, the higher the creativity. In this study, the internal consistency coefficient of the scale was 0.894, and the KMO value was 0.901.

### Data Analysis

SPSS (version 26.0) and PROCESS plug-in (version 4.0; Hayes, 2021) were used to analyze the data. To ensure the validity of data analyses, Harman's single-factor test using principal component factor analysis was performed to test the common method bias (Podsakoff et al., 2003). The results of unrotated principal component analysis showed that four factors had eigenvalues >1, and their contribution to the total variance was 54.223%. The first factor accounted for only 25.997%, which is far below the critical criterion of 40% (Aguirre-Urreta and Hu, 2019), indicating that there was no significant common method bias. In other words, the variation between the independent and dependent variables was caused more by the difference in the nature of the variables than by the methods of data collection and measurement. Following the test of common method bias, descriptive statistical analysis was performed: the mean and standard deviation of each variable were calculated to observe the trend of concentration and dispersion. Then, Pearson product–moment correlation coefficients were calculated to test the correlation on all variables. Finally, PROCESS plug-in (version 4.0; Hayes, 2021) was used to test the mediation model.

## Results

### Descriptive Statistics and Correlation Analyses

Among the interviewees, 260 (25.072%) were male and 777 (74.928%) were female. As for the residential address, 621 students (59.884%) lived in urban areas and 416 (40.115%) in suburban areas. The descriptive analyses of sense of place, creativity, and academic achievement in geography among the interviewees are summarized in Table 1.

**Table 1:** Descriptive statistics for the three variables.

**Variable**		***N***	**M**	**SD**
Sense of place	1,037		3.370
	Gender			
	Male	260	3.320	0.549
	Female	777	3.387	0.454
	Residential address			
	Urban	621	3.384	0.515
	Suburban	416	3.349	0.423
Creativity	1,037		2.643
	Gender			
	Male	260	2.756	0.748
	Female	777	2.605	0.714
	Residential address			
	Urban	621	2.800	0.751
	Suburban	416	2.409	0.616
Academic achievement in geography	1,037		3.070
	Gender			
	Male	260	3.250	1.238
	Female	777	3.010	1.056
	Residential address			
	Urban areas	621	3.250	1.132
	Suburban areas	416	2.800	1.017

Pearson product–moment correlation coefficients were computed to assess the relations among the three variables. Sense of place was found to be positively and significantly correlated with academic achievement in geography (*r* = 0.196, *p* < 0.001). There was a significant positive correlation between sense of place and creativity (*r* = 0.253, *p* < 0.001). The positive correlation between creativity and academic achievement in geography (*r* = 0.341, *p* < 0.001) was also significant. The correlation results are presented in Table 2.

**Table 2:** Pearson's *r* for the three variables.

**Variable**	**Sense of**	**Creativity**	**Academic achievement**
	**place**		**in geography**
Sense of place	1		
Creativity	0.253^***^	1	
Academic achievement	0.196^***^	0.341^***^	1
in geography			

*** *p < 0.001*.

### Mediation Analysis

The PROCESS plug-in (version 4.0; Hayes, 2021) was used to perform mediation analysis, with sense of place as the independent variable, academic achievement in geography as the dependent variable, and creativity as the mediation variable (Model 4). Based on the results of the literature review, the two most frequently reported factors affecting the dependent and mediation variables—gender and residential address—were treated as covariates. The results (see Table 3) indicate that sense of place significantly predicts academic achievement in geography (β = 0.452, *t* = 6.566, *p* < 0.001), and the prediction remains significant even when creativity is entered (β = 0.296, *t* = 4.307, *p* < 0.001). Moreover, sense of place was found to positively predict creativity (β = 0.378, *t* = 8.660, *p* < 0.001). Furthermore, creativity had a positive effect on academic achievement in geography (β = 0.411, *t* = 8.677, *p* < 0.0001). In addition, both the direct effect of sense of place on academic achievement in geography and the mediating effect of creativity had bootstrap confidence intervals (95%), with no zero between their lower and upper limits (see Table 4). This suggests, after controlling for gender and residential address, that sense of place can directly predict academic achievement in geography and also predict it indirectly *via* creativity. The direct effect (0.296) and the mediation effect (0.155) accounted for 65.708 and 34.292% of the total effect, respectively. Definitely, our findings about the mediating role of creativity may be only partial, demanding further attention.

**Table 3:** Mediation analysis results for the three variables.

**Regression equation**		**Fitting indices**				**Significance**
**Outcome variable**	**Predictor variable**	***R***	***R* ^2^**	***F*** **(df)**	**β**	**t**
Creativity		0.374	0.140	56.015^***^		
	Gender				−0.172	−0.197^***^
	Residential address				−0.376	0.625
	Sense of place				0.378	8.660^***^
Academic achievement in geography		0.385	0.149	45.010^***^		
	Gender				−0.198	–.321
	Residential address				−0.277	−4.113^***^
	Creativity				0.411	8.677^***^
	Sense of place				0.296	4.307^***^
Academic achievement in geography		0.294	0.086	32.572^***^		
	Gender				−0.269	−3.528^***^
	Residential address				−0.431	−6.412^***^
	Sense of place				0.452	6.566^***^

*** *p < 0.001*.

**Table 4:** Total effect, direct effect, and indirect effects among the variables.

**Effect**	**Effect size**	**Boot SE**	**Boot CI lower limit**	**Boot CI upper limit**	**Relative effect size**
Total effect	0.452	0.069	0.317	0.587	
Direct effect	0.297	0.069	0.161	0.432	65.708%
Indirect effect	0.155	0.029	0.103	0.215	34.292%

*Boot SE, boot standard error; boot CI, boot confidence interval; lower limit and upper limit, lower limit and upper limit of 95% boot confidence interval; relative effect size, direct effect size or indirect effect size divided by the total effect size*.

**Figure 2:**
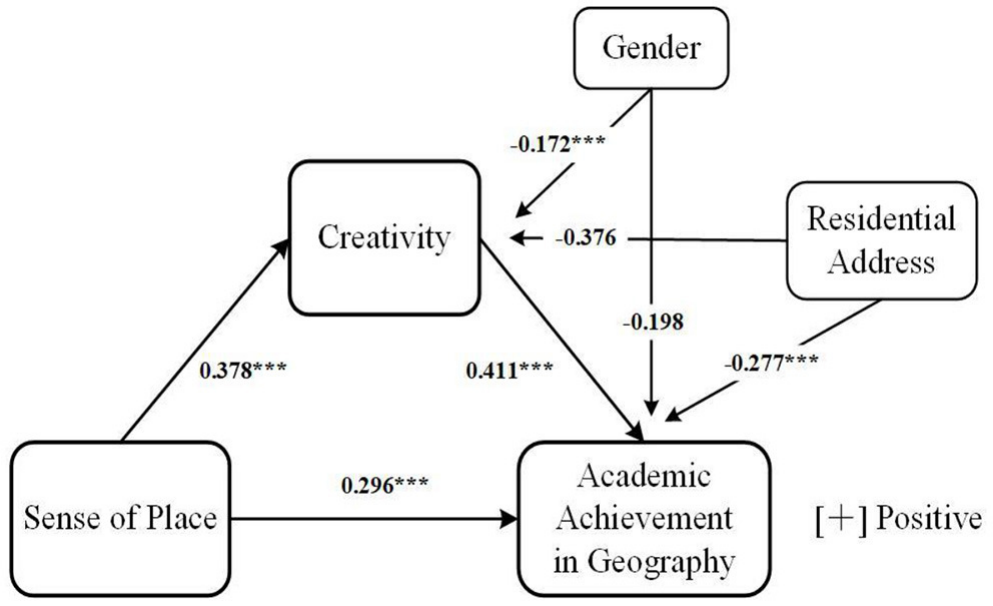
Relationships validated in the study. *** *p* < 0.001.

In addition, the results (see Table 3) showed that gender contributed to the variance in creativity (β = −0.172, *t* = −0.197, *p* < 0.001). Compared with girls, the creativity of boys is higher. When the association between sense of place and academic achievement in geography was tested, both gender (β = −0.269, *t* = −3.538, *p* < 0.001) and residential address (β = −0.431, *t* = −6.412, *p* < 0.001) had a remarkable effect on academic achievement in geography. Moreover, the residential address was still significant even when creativity was entered into the model (β = −0.277, *t* = −4.113, *p* < 0.001). We found students from suburban areas have lower academic achievement in geography. Figure 2 provides a graphic representation of these relationships.

## Discussion

### Discussion of Results

In this study, we created a mediation model that indicates the relationship between sense of place and academic achievement in geography, as well as the mediating role of creativity. The results of this study are congruent with the hypotheses proposed and with previous research.

First, these results are consistent with Hypothesis 1 and also with the previous studies, suggesting that sense of place positively relates to academic achievement in geography. For example, some studies showed that a cohesive, supportive, and equitable learning environment can improve academic achievement in geography (Chionh and Fraser, 2009; Schultz and DeMers, 2020; Jochecová et al., 2022). Also, positive perceptions and experiences from the surrounding environment promote academic achievement in geography (Likouri et al., 2017). Findings from neuroscience research also support this result: an fMRI study suggested that perceiving a change from the environment improves behavior, which exerts a positive effect on academic achievement (Wixted et al., 2016). In addition, consistent with our expectations, affection and emotion can improve academic achievement in geography (Hill et al., 2021). For instance, students who feel pride in the geography learning process perform better than those who do not (Ye et al., 2021a). An eye-tracking experiment revealed that more positive and profound emotional experiences boost comprehension of geographic concepts (Wang et al., 2021). All these findings indicate that sense of place is a combination of the environment and perception, as well as an emotional connection between humans and places. Sense of place can offer physical and mental support from the environment, positively influencing academic achievement in geography.

In addition, the development of sense of place is especially related to the place where a person lives (Steele, 1981). The place has the triple attributes of physics, function, and meaning (Steele, 1981; Bott and Banning, 2008). Quite possibly, a strong sense of place represents an individual's deep insights into the geographic location, functional facilities, and social culture of the surrounding environment (Larson et al., 2013). “Geography for life” is an important characteristic of geography education (Bednarz, 1994; Heffron, 2012; Meadows, 2020). In particular, embodied experiences from daily life have been confirmed as an important foundation and useful resource for geography learning (Nairn, 2005; Israel, 2012; France and Haigh, 2018; Kim, 2019). Therefore, place dependence, attachment, and identity could affect students' perceptions, cognitions, experiences, and affections with regard to geographical environments, thus contributing to the improvement in academic achievement in geography.

Second, the results are consistent with Hypothesis 2, as well as with other studies: as predicted, these studies reveal that sense of place positively influences creativity. Evidence from the investment theory of creativity (Sternberg and Lubart, 1991) and the 4P model of creativity (Rhodes, 1961) matches our hypotheses. Creativity is generally considered to be the result of the interaction between individuals and the environment. Some studies corresponded natural and social environments with creativity, revealing that positive environments improve creativity (Kandler et al., 2016). Moreover, psychological evidence has suggested that creative behavior is related to sensory stimulation and perceptual system (Maslow, 1962). Also, positive perceptions and emotions can significantly enhance creativity (Mottron et al., 2009; Necka, 2011; Xu et al., 2020; Csizmadia et al., 2021; MacGregor and Joshi, 2021). Positive emotions trigger spontaneous brain activities and stimulate creative thinking, according to a resting-state fMRI study (Shi et al., 2018). These findings show that a strong sense of place, characterized by affective interaction between humans and places, exerts a positive influence on creativity. Furthermore, sense of place, as a combination of the environment and perception, is closely related to perception and emotion (Tuan, 1975). As we noted before, the stronger the sense of place, the more the dependence on, attachment to, and identity with place (Low and Altman, 1992; Kyle and Chick, 2007). Sense of place may augment the relationship between students and their surrounding environment, particularly through perception and emotion, increasing the possibility of creative activity. In addition, the ideas of the 4C model of creativity (Kaufman and Beghetto, 2009) are consistent with our hypothesis. Research on mini-c and little-c creativity has suggested that they occur in nearby environments like school and hometown, potentially transforming into pro-c and big-C (Jones, 2011; Grohman, 2018). Therefore, we can reasonably conclude that both sense of place and creativity are essentially related to cognitive activities, which share a common location of occurrence (environment), medium of occurrence (perception, affection, etc.), and mechanism of occurrence (human–place interaction).

Third, the results validated Hypothesis 3, showing that creativity positively affects academic achievement in geography, which is consistent with other studies. Creativity as an individual trait is an important foundation for learning in school, as well as for future work and even life-long development (Runco, 2008). According to the literature, students with a higher level of creativity can attain higher academic achievement in geography. Novelty and validity are two features of creativity, which can be explained using the following formula (Amabile et al., 1996; Kaufman and Beghetto, 2009; Simonton, 2012; Sternberg, 2012; Gajda et al., 2017):


$$\begin{array}{l} C=O*TC \end{array}$$

Here, C stands for creativity, O represents originality, and TC is defined as task constraints (Gajda et al., 2017), which demonstrates that creativity is a multiplicative combination of originality and task constraints, requiring not only original and divergent thinking (Dunbar, 1997) but also completing specific tasks that involve deduction, reasoning, and practice (Kaufman et al., 2016b). Extant research acknowledges that divergent thinking and task completion capabilities are closely related to academic achievement, supporting the view that creativity positively predicts academic achievement (Bentley, 1966; McClelland et al., 2014; Wilder, 2015; Ulger, 2018; Sun et al., 2020). Furthermore, a study based on the Four-C model of creativity shows that mini-c and little-c creativity are associated with the learning process (Beghetto and Kaufman, 2014). Mini-c, new and meaningful personal insights and ideas that stimulate motivation for and interest in learning, possibly improves academic achievement (Beghetto and Kaufman, 2007). Little-c creativity plays a key role in processes like inquiry-based learning, impromptu speaking, and problem resolution (Ryan and Deci, 2000; Kaufman et al., 2016a). Geography involves multiple ways of thinking, such as scale thinking and spatial thinking (Lambert, 2010; Lambert and Jones, 2017). There is growing evidence that the collection and summarization of geographic information, as well as the selection and application of geographic skills, are linked to students' creativity (Favier and van der Schee, 2012; Collins, 2018). Furthermore, geographical learning has been defined as a creative and imaginative process (Catling, 2005, 2013). Consistent with these arguments, Scoffham (2013) proposed that geography education can be enriched by creative practice. Therefore, it is fair to conclude that creative students possess high levels of geographic competence (which comprises geographic knowledge, skills, and thinking), which usually translates into a high level of academic achievement in geography.

Fourth, the findings are consistent with Hypothesis 4. We found that creativity can play a mediating role between sense of place and academic achievement in geography, revealing a path whereby sense of place influences creativity. To start with, students with stronger sense of place perform more creatively, relating to the support of environments, perceptions, and emotions offered by sense of place (Relph, 1976). Second, creativity is also critical to learning geography (Catling, 2013; Yli-Panula et al., 2019). Based on fMRI investigations, creativity has been linked to the co-participation of cognitive and thinking processes, which is related to multiple areas of the brain (Fink et al., 2010; Boccia et al., 2015; Ye et al., 2021b). Furthermore, evidence from high-resolution EEG studies has shown that the brains of high-level creatives exhibit more right-hemispheric alpha activities than low-level creatives (Arden et al., 2010; Tan et al., 2019). Several studies have proposed that alpha activities can both promote inspiration and accelerate information collection, activities that are widely considered to comprise the optimum neurologic state of learning and thinking (Fiore et al., 2000; Jawed et al., 2019; Harada et al., 2020). Overall, emerging research findings extend comprehension of creativity: people with higher levels of creativity perform better at cognitive processing, problem-solving, and other thinking processes. In other words, creativity strengthens cognitive processes, such as perception and memory, which intensify perception and experience of place, allowing individuals to gain more positive affective power from place and thereby improving academic achievement in geography.

Fifth, the results indicate that the differences in gender predict the variance in creativity. Our finding shows that the creativity of male students in our study is higher, which is in line with several existing studies (Gunawan et al., 2020; Permatasari and Budiyono, 2020; Xu et al., 2022). One possible explanation is male students have a better ability to think and solve problems than female students (Doleck et al., 2017; Abdulla et al., 2020; Zibenberg and Da'as, 2022), influencing the development of creative thinking skills (Hidayat et al., 2018; Rahmawati et al., 2019). Indeed, the unequal distribution in the gender of interviewees may have an impact on this result, deserving further study. The results also indicate that students from urban areas have higher academic achievement in geography, which is consistent with previous studies (Lembani et al., 2020; Solem et al., 2021; Bourke et al., 2022). In fact, there are some studies showing that city life offers more real experience related to geography curriculum or fieldwork (Cook et al., 2006; Wilson et al., 2017), promoting the development of geographical knowledge and skills (Simm and Marvell, 2015).

In the current study, it is worthwhile to note that creativity only partially mediated the relationship between sense of place and academic achievement in geography. Analysis of the data showed that sense of place accounted for most of the variance in academic achievement in geography (65.708%), indicating that the mediating role of creativity was not predominant (34.292%). In other words, although high levels of creativity can influence geography achievement to some extent because of sense of place, sense of place still exerts a significant positive effect on academic achievement in geography.

### Implications

By expanding upon and complementing previous findings regarding academic achievement in geography, this study makes both theoretical and practical contributions. In terms of theoretical implications, this study is unique in specifically linking sense of place to academic achievement in geography, deepening comprehension of its impact on academic achievement. In addition, the mediating and buffering effects of creativity demonstrated suggest that sense of place may enhance creativity, which ultimately improves academic achievement in geography. In terms of practical implications, the relationship between the three variables examined may help geography teachers better understand the mechanisms of academic achievement in geography and the special function of sense of place, particularly leading to greater real-life teaching implications, which helps students improve their academic achievement in that discipline.

### Limitations and Future Directions

Despite its strengths, the present study has some limitations. First, the study is a cross-sectional study, so not enough has been done on longitudinal studies. Second, all the participants were from western regions of China, which might undermine the generalizability of the findings. Third, the imbalance in the gender ratio of the participants may also hamper the generalization of the results. In future, researchers could use a longitudinal survey design to collect data over a period of time and recruit participants equally from different schools in different regions, focusing on the impact of city size and spatial distance. In addition, they could examine which specific aspect of creativity mediates the association between sense of place and academic achievement in geography or which dimension of sense of place significantly influences academic achievement in geography. Although the mechanisms related to academic achievement in geography are still contested, this study may provide empirical evidence and new insights for future researchers.

## Conclusion

This study explored the relationship between sense of place and academic achievement in geography and the mediating effect of creativity between the two. The results suggest that sense of place is a positive predictor of academic achievement in geography and may increase this to some extent. Furthermore, individuals with a high score for creativity achieve better academically in geography than those with low scores for creativity. It is worth noting that although creativity certainly plays a mediating role, the improvement in academic achievement in geography is still mainly attributable to sense of place.

## Data Availability Statement

The raw data supporting the conclusions of this article will be made available by the authors, without undue reservation.

## Ethics Statement

The studies involving human participants were reviewed and approved by The Ethics Committee of Zhejiang Normal University. Written informed consent to participate in this study was provided by the participants' legal guardian/next of kin.

## Author Contributions

YX and JZ designed the research. JZ, JG, YM, ZW, YY, XL, and ZA carried out the literature search and data analysis. JZ, JG, YM, ZW, YY, XL, ZA, and YX wrote the manuscript. All authors have read and agreed to the submitted version of the manuscript.

## Funding

This research was funded by the National Office for Education Sciences Planning (grant number BAA180017).

## Conflict of Interest

The authors declare that the research was conducted in the absence of any commercial or financial relationships that could be construed as a potential conflict of interest.

## Publisher's Note

All claims expressed in this article are solely those of the authors and do not necessarily represent those of their affiliated organizations, or those of the publisher, the editors and the reviewers. Any product that may be evaluated in this article, or claim that may be made by its manufacturer, is not guaranteed or endorsed by the publisher.
